# Therapeutic Effects of Natural Bioactive Compounds in the Management of Human Diseases

**DOI:** 10.3390/cimb47121036

**Published:** 2025-12-12

**Authors:** Sara Spinelli, Alessia Remigante, Rossana Morabito

**Affiliations:** 1 Department of Chemical, Biological, Pharmaceutical and Environmental Sciences, University of Messina, 98166 Messina, Italy; saspinelli@unime.it (S.S.); rmorabito@unime.it (R.M.); 2 Department of Biomedical, Dental and Morphological and Functional Imaging, University of Messina, 98125 Messina, Italy

Over recent decades, scientific interest in natural bioactive compounds has increased considerably, driven by the growing recognition of their therapeutic potential in the prevention and treatment of various chronic diseases. Many of these disorders, including autoimmune and metabolic diseases as well as certain cancers, share a common etiopathogenesis involving redox imbalance and persistent activation of inflammatory pathways. Oxidative stress, resulting from excessive reactive oxygen species (ROS) production, and chronic inflammation are now recognized as key contributors to disease progression, promoting cellular and tissue dysfunction through the aberrant activation of molecular targets such as NF-κB, Nrf2, MAPK, and PI3K/Akt. Natural bioactive molecules derived from plants, algae, and animals have shown remarkable capacity to modulate these signaling pathways through transcriptional, post-transcriptional, and antioxidant mechanisms. Both preclinical and clinical studies support the view that these compounds may represent complementary or alternative therapeutic strategies to conventional pharmacological approaches, owing to their favorable safety profiles and reduced side effects. Nevertheless, further mechanistic insights are required to elucidate the specific molecular interactions underlying their biological activities and to guide the rational development of targeted interventions capable of modulating oxidative and inflammatory processes.

Within this framework, the fifteen contributions collected in this *Special Issue*, including original research articles, reviews, and brief reports, offer an updated overview of the latest advances in the study of natural bioactive compounds. Together, these papers highlight their ability to modulate redox balance and inflammatory signaling, paving the way for the development of innovative nutraceutical and pharmacological approaches based on natural sources.

Below, we summarize the key thematic areas represented in this collection.

**Inflammatory and Autoimmune Disorders**

Several studies highlight natural compounds that attenuate inflammation and regulate immune responses through redox-sensitive pathways:

- Du et al. [1] demonstrated that emodin alleviates rheumatoid arthritis by modulating the HIF-1α/NLRP3 axis and mitochondrial autophagy, reducing pyroptosis and oxidative injury.
- Wang et al. [2] reported that periplosides from *Cortex periplocae* improve collagen antibody-induced arthritis by promoting macrophage M2 polarization, pointing to immunomodulation as a therapeutic mechanism.
- Theodosis-Nobelos et al. [3] reviewed the application of polymeric nanotherapeutics loaded with pleiotropic antioxidants (e.g., curcumin, resveratrol, quercetin) for autoimmune disease management, emphasizing enhanced bioavailability and tissue targeting.
- Sheng et al. [4] showed that alkaloids from Rushanhu (*Zanthoxylum nitidum var. tomentosum*) mitigate rheumatoid arthritis progression through modulation of the SRC/STAT3/MAPK signaling axis, reducing synovial hyperplasia, inflammation, and oxidative damage.

Together, these studies reinforce the interconnected roles of oxidative stress, inflammation, and immune dysregulation in autoimmune disorders, and position natural compounds as promising multi-target therapeutic agents capable of restoring cellular homeostasis.

**Metabolic and Endocrine Regulation**

- Natural compounds are also explored for their ability to restore metabolic balance and counteract high glucose-related oxidative stress:
- Shin et al. [5] found that a fermented *Aralia cordata* extract regulates glucose transporters (SGLT1, GLUT2) and enhances GLP-1 secretion, while promoting Th2 immune responses and antimicrobial peptide production.
- He and Cui [6] reviewed plant heteropolysaccharides with anti-diabetic properties, detailing mechanisms related to insulin sensitivity, β-cell protection, and gut microbiota modulation.
- Mangarov et al. [7] revisited alpha-lipoic acid, a well-established antioxidant used in diabetic peripheral neuropathy, providing a critical appraisal of clinical efficacy and challenges in long-term management.

These papers collectively depict a growing recognition of natural products as regulators of redox-metabolic homeostasis and glycemic control.

**Liver Function and Lipid Metabolism**

The hepatoprotective potential of natural molecules is highlighted in the following contributions:

- Singh et al. [8] examined the action of *Angelica keiskei* extract against hepatocellular carcinoma, combining cytotoxic assays and molecular docking analyses.
- Sabini and Timotius [9] reviewed curcumin’s ability to mitigate lipid accumulation, oxidative stress, and fibrosis in metabolic dysfunction-associated steatotic liver disease (MASLD).

These contributions underscore the dual antioxidant and metabolic regulatory role of natural products in liver health and disease prevention.

**Marine and Algal Bioactives**

Marine organisms emerge as valuable sources of anti-inflammatory agents:

- Lee et al. [10] showed that extracts from the green alga *Caulerpa okamurae* suppress NF-κB activation and nitric oxide production in macrophages exposed to *Porphyromonas gingivalis*.
- Kim and Kim [11] demonstrated similar anti-inflammatory properties of *Apostichopus japonicus* (sea cucumber) extract, suggesting applications in oral and systemic inflammatory disorders.

These findings highlight the marine environment as an untapped reservoir of bioactive molecules with immunomodulatory and antioxidant properties.

**Oxidative Stress and Redox Homeostasis**

A core conceptual axis of this Special Issue is the role of redox regulation in health and disease:

- Spinelli et al. [12] reviewed the mechanisms of redox homeostasis in red blood cells, detailing how antioxidant systems preserve membrane integrity and discussing novel antioxidant strategies.
- Yan et al. [13] provided a complementary perspective by characterizing the antioxidant and pharmacological profiles of *Citrus grandis* essential oils through GC-MS/MS and network pharmacology.
- Together, these papers reaffirm oxidative stress as a unifying mechanism underlying multiple pathologies.

**Cancer and Molecular Targeting**

Natural products as anticancer agents are discussed by Alsharairi et al. [14], who examined licorice chalcones as modulators of gene expression and signaling pathways in nicotine-induced non-small-cell lung cancer, pointing to their role in mitigating tobacco-related carcinogenesis.

**Antimicrobial and Translational Perspectives**

Wali et al. [15] offered a broad review of natural biomolecules with antimicrobial and antifungal activity, integrating emerging microbial management technologies and bioengineering approaches for drug discovery.

Taken together, the articles included in this Special Issue illustrate the rich therapeutic potential offered by natural bioactive compounds. From marine organisms to medicinal plants, and from classical antioxidants to innovative nano-formulations, these studies deepen our understanding of the molecular targets and mechanisms underlying the beneficial effects of natural molecules on human health. The collected evidence supports the view that natural compounds are not merely complementary or alternative treatments, but rather scientifically validated modulators of pathological pathways, particularly those involved in oxidative stress, inflammation, metabolic dysregulation, and immune dysfunction. We hope that this Special Issue will inspire further mechanistic research, translational applications, and clinical investigations, ultimately contributing to the development of multi-target therapeutic strategies that minimize the side effects often associated with conventional drugs.
